# Genome-wide detection of hybrid genes with multiple components in human

**DOI:** 10.1186/1756-0500-2-75

**Published:** 2009-05-06

**Authors:** Yun-Huei Tzeng, Sheng-Shun Wang

**Affiliations:** 1 Genomics Research Center, Academia Sinica, Taipei, Taiwan; 2 Department of Computer Science and Information Engineering, National Taiwan University, Taipei, Taiwan

## Abstract

**Background:**

Previous studies showed that gene hybrid is one of the principal processes for generating new genes. Although some gene hybrid events have been reported to be inter- or intra-species, there lacks a well-organized method for large scale detection of the events with multiple components. Hence in this study, we focus on building up an efficient method for exploring all candidates of gene hybrid events in human genome and provide useful results for further study.

**Findings:**

We have developed a method designated Triad Comparison Algorithm (TCA) to detect all potential *N*-hybrid events (i.e., an *N*-hybrid gene and its *N *non-overlapping component regions derived from *N *different genes) in human genome. The results reveal that there are many convoluted *N*-hybrid events with multiple components (*N *> 2) and that the most complicated *N*-hybrid genes detected in human by TCA are composed of six component regions. Interestingly, our results show that most of the hybrid events belong to the 3-hybrid category. Furthermore, we observe that a single gene might participate in different events. Twelve genes were found to have dual identities contained in different *N*-hybrid events (i.e., they were identified as hybrid genes as well as component genes). This points out that to a certain extent the gene hybrid mechanism has generated new genes during the course of human genome evolutionary history.

**Conclusion:**

An efficient method, TCA, is developed for exploring all candidates of hybrid genes in the human genome and provides useful results for the evolutionary analysis. The advantage of TCA is its power of detecting any kinds of hybrid events in any species with a large genome size.

## Background

The emergence of new genes is fundamental to the evolution of lineage- or species- specific traits [1]. Duplication of chromosomal segments provides abundant raw material for the formation of new genes [2,3]. In addition to the gene duplications that have been identified in different scales, recent studies demonstrated that the fusion/fission mechanism may also play an important role in enrichment of new genes and/or genes with multiple protein domains in various species [1,4-12]. For example, most of the proteins in SCOP [13] or Pfam [14] databases harbor two or more domains resulted by a wide variety of domain combinations [15,16]. Moreover, multiple functional domains in proteins have been considered as essential units for the modular assembly of new genes [17-21]. It has been shown that gene hybrid events across genomes can be used in predicting functional associations of proteins, including physical interactions and complex formations. This prediction relies on an observation that two proteins functioning in the same complex in one organism frequently fused into a single "Rosetta Stone" protein in another organism (i.e., "Rosetta Stone" protein deciphers the interaction between the protein pairs) [7,22]. A previous study reported that the monkey king gene family of *Drosophila melanogaster *was originated from retroposition followed by gene fission event [9].

Recently, the gene fusion/fission has been demonstrated to largely contribute to the evolution of multi-domain proteins in bacteria [23]. From literature, some systematic methods have been proposed for detection of gene hybrid events [6,12,22,24-26]. The methods are not very efficient for genome-wide detection of hybrid events with arbitrary number (*N*) of components because they are based on pair-wise sequence comparison. Therefore, their computational complexity will increase exponentially as *N *increases, leading to a difficulty of the large scale detection, especially for multiple components.

In this study, we have built up an efficient method for exhaustively exploring all candidates of gene hybrid events (for all possible *N *components, where *N *≧ 2) with flexible criteria in the human genome. This result reveals that some of the hybrid events are complicated and that some genes seem to undergo such the events multiple times in the human genome.

## Methods

**Figure 1:**
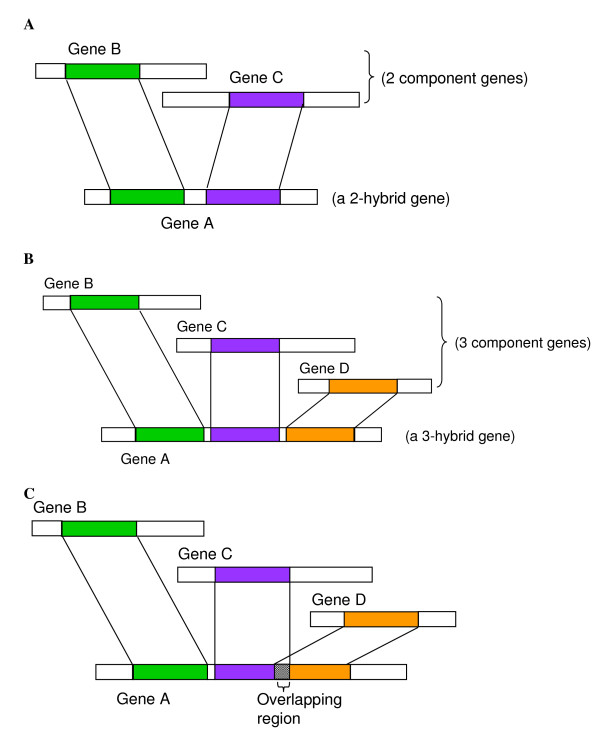
**Examples of targeting result in this study**. (a) a model example of a 2-hybrid event (b) a model example of a 3-hybrid event (c) an example that will be rejected in 3-hybrid event detection because of the overlapping of two component regions.

We propose a progressive algorithm, Triad Comparison Algorithm (TCA), to identify *N*-hybrid events in human genome. Each event is composed of an *N*-hybrid gene and *N *component genes (*N *= 2). An *N*-hybrid gene is a gene that contains *N *non-overlapping component regions derived from *N *different genes (i.e., the "*N *component genes"). These *N *component genes are defined as being not alignable with each other. Here, we also term a 2-hybrid event as a "triad", which is the basic unit for identifying all other *N*-hybrid events (*N *> 2) in the genome studied. A model of a triad is illustrated in Figure 1A, which depicts that a 2-hybrid gene "Gene A" is derived from two component genes "Gene B" and "Gene C". An example of 3-hybrid event is shown in Figure 1B. Moreover, Figure 1C shows an example that will be rejected in 3-hybrid event detection because of the overlapping of two component regions.

**Figure 2:**
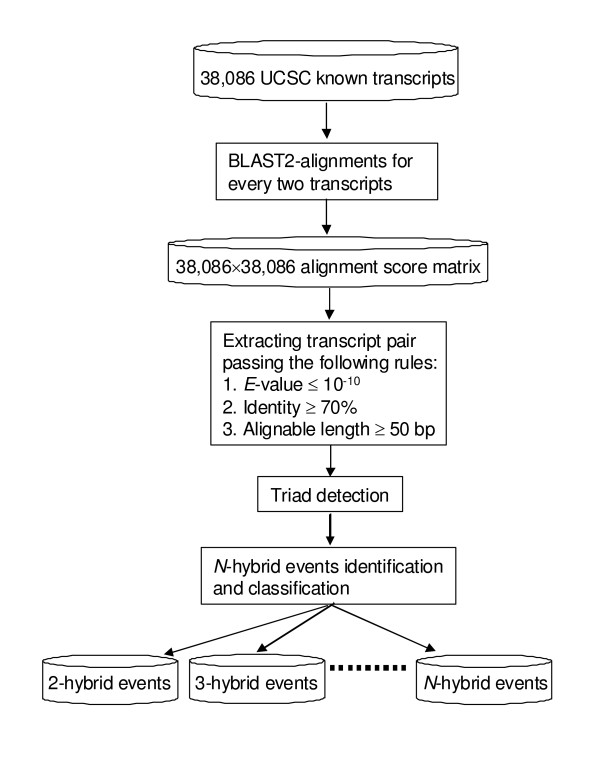
**Flowchart of the study**.

We download the known-Isoforms file (HG17) of human genes from UCSC website . There are 20,181 groups for 38,086 transcript IDs and all isoform groups are different from each other. We used BLAST(2.2.11) program from NCBI to do the pair-wise comparison for all transcript sequences. After collecting the alignment score matrix (including sequence IDs, *E*-value, identity, and aligned region etc.), we set up filtering criteria (length of component regions from 50 to 150 bp, *E*-value < 10^-10^, identity > 70%) to define the relatedness between gene pairs and used TCA to detect all triads (2-hybrid events). Based on the triads, we can further search *N*-hybrid events with *N *> 2. The flowchart of this study is shown in Figure 2. For the detailed description of TCA, please see the Additional File 1.

## Results and discussion

### Complicated *N*-hybrid Events Have Longer Component Regions

Because different transcripts may contain different regions in gene fusion/fission events [9], the purpose of this study is to detect the *N*-hybrid events in transcription sequence level. The summary of all detected results are shown in Table 1. To understand the degree of component genes that have contributed to *N*-hybrid events (*N *= 2, 3, 4, 5, and 6), we calculate the length proportion of components to hybrid gene for each *N*-hybrid event. Because the tendency is similar for all length criteria of components, we present the results with 50 and 100 bp in Table S1 (Additional File 2).

**Table 1:** The number of detected *N*-hybrid events under different length criteria of components (identity > 0.7 and *E*-value < 10^-10^)

	# of *N*-hybrid events				
	
Length of component regions (nucleotides)	2	3	4	5	6
50	438	701	105	34	14
60	253	700	105	34	14
70	228	870	8	1	0
80	144	835	8	1	0
90	135	703	8	1	0
100	135	703	8	1	0
110	79	575	0	0	0
120	57	434	0	0	0
130	53	259	0	0	0
140	56	205	0	0	0
150	43	176	0	0	0

The proportions of component regions between different *N*-hybrid events are significant different (*P *< 0.001, Kolmogorov-Smirnov Test). Table S1 shows that the 3-hybrid event has the smallest proportion, on average, came from the corresponding component genes under all different length criteria. Although each component region contained in 3-hybrid genes usually has longer length than that contained in 2-hybrid genes, but on average the length of 3-hybrid genes is even longer than that of 2-hybrid genes. This conduces that 3-hybrid event has the smaller proportion of component regions than 2-hybrid event. When the length criteria of component regions are larger than 100 nucleotides, only few *N*-hybrid events with *N *= 4 and 5 have been found (Table 1). For these 4- and 5-hybrid events, their proportions of component regions are larger than 3-hybrid events on the whole.

### One Gene Could Participate in Different *N*-hybrid Events

In the results, different *N*-hybrid events may have the same hybrid gene or part of component genes. In Table S2, we count the number of different hybrid genes and component genes contained in all *N*-hybrid events with each component length is larger than 50 bp (Additional File 3).

**Figure 3:**
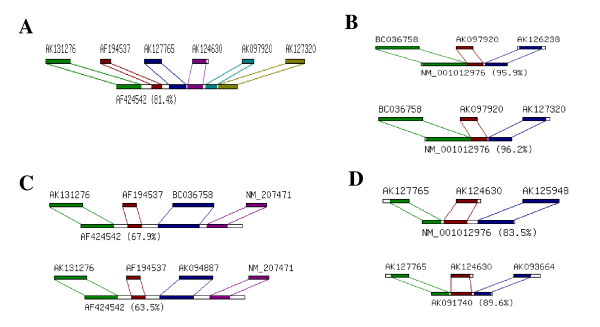
**Examples of one gene can be involved in different *N*-hybrid events**. The percentage followed by the *N*-hybrid gene is the ratio of all components contained in the hybrid gene. (a) a 6-hybrid event in the human genome (b) an example of two 3-hybrid events that differ by only one component gene (c) an example two 4-hybrid events that differ by only one component gene (d) an example of two 3-hybrid events with two common component genes but different hybrid genes. The percentage showed in figures means the proportion of all component regions contained in the *N*-hybrid gene.

There are 2- to 6-hybrid events contained in Table S2 and the most of the events belong to the 3-hybrid category. The most complicated events belong to the 6-hybrid category and we present one of them in Figure 3A. If we only take account of the different hybrid and component genes, the numbers are much smaller than those of *N*-hybrid events listed in Table 1. This is because same genes could be contained in different *N*-hybrid events. Selected cases of this kind of events are displayed in Figure (3B, C, and 3D).

Figure 3B indicates that NM-001012976 is a 3-hybrid gene with two possible combinations of three component genes: (BC036758, AK097920, AK126238) and (BC036758, AK097920, AK127320). The two combinations are different only in one of the component genes, AK126238 and AK127320, which belong to different isoform groups. The similar phenomenon can also be found in some 4-hybrid events (Fig. 3C). The 4-hybrid gene AF424542 has two possible combinations of four components: (AK131276, AF194537, BC036758, NM_207471) and (AK131276, AF194537, AK094887, NM_207471), that have three common component genes.

There are some more complicated cases. Figure 3D shows that two genes, AK127765 and AK124630, both are components of two different 3-hybrid genes, NM_001012976 and AK091740. Another example is shown in Figures 3B and 3C, which a component gene, BC036758, contribute to a 3-hybrid gene, NM_001012976, and a 4-hybrid gene, AF424542, as well. Similarly, the events in Figure 3C (4-hybrid event) and Figure 3A (6-hybrid event) have two common component genes, AK131276 and AF194537.

### Multiple Origins of Hybrid Genes in Human

In the results, we have found 12 genes with dual identities, which can be identified as a component gene or a hybrid gene in different hybrid events. For simplicity, we term them "mixed Rosetta Stone" (MRS) genes. They are listed in Table S3 (Additional File 4) and all corresponding events can be found in Additional File 5 and Additional File 6. All the MRS genes are contained in very alike events and usually have some similar component genes.

Proteins that were fused into a single "Rosetta Stone" protein frequently function in the same complex and are involved in the same interaction network [7,22]. The MRS genes detected in the human genome strongly indicate functional associations of these proteins, including physical interactions and complex formation. This also reveals that multiple occurrences of gene hybrid mechanism and complex network connection between MRS genes. An example for the existence of complicated hybrid gene network could be found in human neoplasia that acquired gene fusions play a causal role in the initiation of the neoplastic process either by activating proto-oncogenes or creating hybrid genes [27]. Hence the origins of the MRS genes are worth further study in disease research.

### "3" is fundamental?

Remarkably, we detected that the majority of hybrid events are 3-hybrid regardless of the component length criteria used (Table 1), while a small proportion of *N*-hybrid events are found with *N *> 3. This might indicate that the hybrid mechanisms in the human genome are largely involved with at least three genes. According to previous literatures, hybrid genes can be used for predicting functional associations of proteins, including physical interactions and protein complex formation [7,22,23]. For gene-fusion events studied across four genomes – *Escherichia coli*, *Haemophilus influenzae*, *Methanococcus jannaschii*, and *Saccharomyces cerevisiae*, more than 2 (~2.44) proteins, on average, are involved in each fusion event [4]. Furthermore, the recent studies on the protein-protein interaction network in yeast have shown that the median connectivities of networks from various databases are 3 (while the mean connectivities are varied from 4.11 to 6.61) [28,29]. If we treated the relatedness defined in our study as a type of connections between genes, it may be the reason that the most detected *N*-hybrid events are 3-hybrid in the human genome.

We propose a hypothesis that 3-hybrid event is the main composition of gene hybrid mechanism in the human genome. The complete human gene network based on their functional roles is not currently available, yet our preliminary analysis for gene hybrid events still gives some insights on how human genes produced by hybrid mechanism. The study for the demonstration will proceed in the future.

## Conclusion

In this study, we have developed a method, TCA, for the detection of all potential *N*-hybrid events in the human genome. Our result reveals that the hybrid mechanism between genes is an important way to generate new genes in this genome. Furthermore, the results also reveal that one gene could be involved and play opposite roles in different hybrid events. This phenomenon suggests the possibility of multiple occurrences of hybrid mechanism in human genome evolution. It further suggests that the hybrid mechanism is not just an accidental event but an on-going process in the human genome evolution. Another important insight from our results is that the 3-hybrid events may be the basic unit in the complicated hybrid gene network.

## Competing interests

The authors declare that they have no competing interests.

## Authors' contributions

YHT conceived the study and prepared the manuscript. YHT and SSW contributed computational concepts and analyzed the data. All authors read and approved the final manuscript.

## Supplementary Material

Additional File 1 **Triad Comparison Algorithm**. The detailed description the algorithm and the method of detection for all potential *N*-hybrid events in human genome. Click here for file

Additional File 2 **Table S1**. The average length of a single component region, *N*-hybrid gene, and the proportion of all components contained in *N*-hybrid genes under different length criteria (50 and 100 bp). Click here for file

Additional File 3 **Table S2**. The number of events, *N*-hybrid genes, and component genes of all *N*-hybrid events with each component length > 50 nucleotides. Click here for file

Additional File 4 **Table S3**. List of all mixed Rosetta Stone (MRS) genes and some examples of the involved hybrid events with dual identities. Click here for file

Additional File 5 **All *N*-Hybrid Events**. The detailed information of all detected *N*-hybrid events by using the alignment parameters of *E*-value < 10^-10^, identity > 70%, and alignment length > 50 bp between component regions of *N*-hybrid genes and their corresponding component genes. Click here for file

Additional File 6 **All Images**. Graph representations of all *N*-hybrid events in the **Additional file **5. Click here for file
