# Supplementary material for: Genome-wide detection of hybrid genes with multiple components in human
Source: BMC Res Notes. 2009 May 6;2:75. doi: 10.1186/1756-0500-2-75 (PMC2684099; doi:10.1186/1756-0500-2-75)
Supplement: Additional File 1 — Triad Comparison Algorithm. The detailed description the algorithm and the method of detection for all potential N-hybrid events in human genome. [file 1756-0500-2-75-S1.pdf]

**Triad Comparison Algorithm (TCA)**

The known-Isoforms file (HG17) of human genes from UCSC website were aligned with BLAST [1] to identify genes that have an asymmetrical triangular relationship. Figures 1 and 2 show the complete searching models and the flowchart in this study, respectively. To explain TCA, we start from the simplest hybrid event (a 2-hybrid event) detection in the following.

Gene A is a '2-hybrid gene' if gene A is a hybrid gene with two different components from other two genes, namely B and C. For detecting all 2-hybrid genes, we sought to identify all triad genes (B, A, C) such that A and B are related, A and C are related, but B and C are unrelated. These relationships are written as B-A-C, which is called a "triad". The symbol "-" means the relatedness, which is defined in the following section. For this B-A-C notation, the middle gene (A) is a hybrid gene and the others (B and C) are the different component genes. The order of the component genes (B-A-C or C-A-B) is dependent on the positions of component gene sequences in the hybrid gene.

Relatedness is determined by setting criteria on BLAST results with an *E*-value (expectation value) less than a threshold of $10^{-10}$ and filtering criteria for the length (from 50 to 150 bp) and identity (> 70%) for the overlapping segment. The expectation value (*E*-value) is the number of different alignments with scores equivalent to or better than *S* (the score of the alignment pairs that can not be improved by extension or trimming) that are expected to occur in a database search by chance. The score of the alignment pairs is more significant if the *E*-value is lower. The relatedness level for expectation value ($E < 10^{-10}$) was chosen according to the identification of the duplicated genes [2]. The identity for the overlapping segment was chosen based on the distribution of human-mouse ortholog similarities [3].

For the strict necessities for all *N*-hybrid genes ($N \geq 2$), we also require the different components in hybrid gene (i.e., the contributed components come from un-related gene pair (B, C) in the above case) can not be aligned in the BLAST report. The purifying criteria will be automatically applied after detecting an *N*-hybrid gene. If two genes have *E*-value lower than the threshold and pass the filtering criteria, they are called "related".

The algorithm for detecting a "triad" is described in the following. Let $m_{ij}$ = the relatedness function between gene *i* and *j* and is defined by

$$m_{ij} = \begin{cases} 1, & \text{if } (i, j) \text{ is related} \\ 0, & \text{if } (i, j) \text{ is un-related} \end{cases}$$

For the implication of the function $m_{ij}$, we can define the necessary and sufficient conditions for a "triad" is $m_{ij} + m_{ik} + m_{jk} = 2$, for arbitrary three genes *i*, *j*, and *k*. Note that $m_{ij}$ is symmetrical (i.e., $m_{ij} = m_{ji}$). Furthermore, we can distinguish which gene is the middle gene from different $m_{ij}$ values in our result:

$$triad = \begin{cases} i-k-j \text{ or } j-k-i, & if\ m_{ij} = 0 \\ i-j-k \text{ or } k-j-i, & if\ m_{ik} = 0 \\ j-i-k \text{ or } k-i-j, & if\ m_{jk} = 0. \end{cases}$$

Again, the middle gene is the 2-hybrid gene with two other component genes. The order of triads (e.g. $i-k-j$ or $j-k-i$) is dependent on the sequence of contributed components in the middle hybrid gene.

## *N*-hybrid events detection by TCA

For the hybrid gene with *N* component genes (*N* > 2), it is called an "*N*-hybrid" gene. With the same idea in the "triad" detection, we also use relatedness function $m_{ij}$ between gene pairs. For each gene *i*, we collect all other genes *j* with $m_{ij} = 1$ and found the appropriate *N* component from the list of *j*. Hence, the generalized necessary condition for an *N*-hybrid event is $\sum_{i<j} m_{ij} = N$. The number *N* will be increased one by one till there's no fitting *N*-hybrid events can be detected.

In simplified notation, we use the $N$-polygon to represent the detected $N$-hybrid events (i.e., $N$-hybrid gene is in the center and surrounded by its $N$ component genes). For example, for $N = 3$, the graph representation is

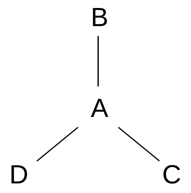


Similarly, the middle gene A is a 3-hrbrid gene with three components coming from genes B, C, and D. For $N = 4$, the graph representation is

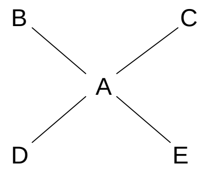


and so on.

From the graph representation, we can observe that each $N$-hybrid event is also an $M$-hybrid event for any $M < N$ according to the definition. For example, an $N$-hybrid event will contain $N$ cases with ($N$-1)-hybrid, $N$($N$-1)/2 cases with ($N$-2)-hybrid, and so on. Obviously, these cases should be excluded from $M$-hybrid events for any $M < N$. Hence, we give a standard for a unique $N$-hybrid event: either the hybrid gene or one of component genes should be different from any other events. Based on the standard, we will delete the repetitions of $M$-hybrid events ($C_N^M$) from $N$-hybrid ones for any $M < N$. Any $N$-hybrid events would be unique in the final results after purifying.

**Computational Identification of *N*-hybrid Events by TCA**

In principle, TCA uses BLAST2-alignments (NCBI) for every two UCSC (University of California, Santa Cruz) known transcripts to identify $N$-hybrid ($N \geq 2$) events (including hybrid genes and component genes) in the human genome. The TCA flowchart is illustrated in Figure 2. Based on the UCSC-identified transcripts

(38,086 different transcripts), we first use the BLAST2 alignment to generate a 38,086×38,086 alignment score matrix, which contains the alignment results of 38,086×(38,086-1)/2 different transcript pairs. The recorded alignment score of a transcript pair includes $E$-value (expectation value), identity, and alignment length is used as criteria to retrieve candidates from all the transcript pairs. We only consider the transcript pairs whose alignment score satisfies the following criteria: $E$-value < $10^{-10}$, identity > 70%, and alignment length > 50 bp.

A total of 688 transcript pairs (202 different transcripts) were identified under the above criteria. We then extracted 796 triads (2-hybrid events), i.e., one hybrid gene derived from 2 component genes, from these 688 alignable transcript pairs. Figure 1A shows an example of a triad "B-A-C", which is composed of a 2-hybrid gene (gene A) and two non-alignable component genes (genes B and C). At this stage, for each $N$-hybrid event, $N$×($N$-1)/2 triads will be selected by this detection. For example, for the 3-hybrid event shown in Fig. 1B, three triads "B-A-C", "B-A-D", and "C-A-D" could be identified at the process. Such an example is illustrated in Figure 1C, in which gene A contains three component regions separately derived from genes B, C, and D; meanwhile, genes C and D are partially overlapped. In this case, only two triads B-A-C and B-A-D are identified by TCA. For each triad, because the two component genes/transcripts are non-alignable, the two component genes/transcripts are very unlikely duplicated or belong to the same alternatively spliced gene group.

Subsequently, we extract 2-, 3-,…, $N$-hybrid ($N \geq 2$) events from the 796 triads identified. In this study, we identify 438 cases with 2-hybrid, 701 cases with 3-hybrid, 105 cases with 4-hybrid, 34 cases with 5-hybrid, and 14 cases with 6-hybrid (Table 1) by using the alignment parameters of $E$-value < $10^{-10}$, identity > 70%, and alignment length > 50bp between component regions of $N$-hybrid genes and their corresponding component genes. We present the details and images of all detected $N$-hybrid events in the supplementary file. The maximal $N$ detected is six in the human genome. Note that, subsets of an $N$-hybrid event cannot be counted in any $M$-hybrid ($M < N$) events repeatedly. For example, the event shown Figure 1B is counted as a 3-hybrid event. Subsets of the 3-hybrid event such as "B-A-C", "B-A-D", and "C-A-D" must be excluded from the counts of 2-hybrid events. In addition, the case illustrated in Figure 1C is regarded as two 2-hybrid events ("B-A-C" and "B-A-D") but not a 3-hybrid event because genes C and D are alignable. Under the same alignment parameters of $E$-value and identity, the distributions of different alignable lengths (60~150bp) are also listed in Table 1. We find that the number of $N$-hybrid events is not stringently

decreased as $N$ increases, because the majority cases belong to $N = 3$ (Table 1). The tendency holds well regardless the parameter of alignable length.

We also find that the number of $N$-hybrid events is generally decreasing as the length of component regions increases in most of cases (Table 1). But there are two exceptions in our identification — one for 3-hybrid detection (lengths of the detected component regions from ≥ 60 to ≥ 70 bp) and the other for 2-hybrid detection (from ≥ 130 to ≥ 140 bp). By increasing the length of component genes from 60 to 70 bp, the number of 3-hybrid genes increases from 700 to 870 while the number of 4-hybrid genes decreases from 105 to 8. This means the length of the majority of sequences contributed by component genes in 4-hybrid evens are ranging between 60 to 70 bp. Hence when the length criteria are raised from 60 to 70 bp, most of the 4-hybrid events are not indentified for length criterion of 70 bp and most might be classified into other lower $N$-hybrid events (e.g. 3-hybrid). In deed, we observe a remarkable increase in the number of 3-hybrid and decrease in that of 4-hybrid events when the length criteria are changed from 60 to 70 bp (Table 1).

**Reference:**


1. Tatusova TA, Madden TL: **BLAST 2 Sequences, a new tool for comparing protein and nucleotide sequences**. *FEMS Microbiol Lett* 1999, **174**(2):247-250.
2. Gu Z, Steinmetz LM, Gu X, Scharfe C, Davis RW, Li WH: **Role of duplicate genes in genetic robustness against null mutations**. *Nature* 2003, **421**(6918):63-66.
3. Veeramachaneni V, Makalowski W, Galdzicki M, Sood R, Makalowska I: **Mammalian overlapping genes: the comparative perspective**. *Genome Res* 2004, **14**(2):280-286.
