# Supplementary material for: Genome-wide detection of hybrid genes with multiple components in human
Source: BMC Res Notes. 2009 May 6;2:75. doi: 10.1186/1756-0500-2-75 (PMC2684099; doi:10.1186/1756-0500-2-75)
Supplement: Additional File 2 — Table S1. The average length of a single component region, N-hybrid gene, and the proportion of all components contained in N-hybrid genes under different length criteria (50 and 100 bp). [file 1756-0500-2-75-S2.pdf]

Table S1. The mean (median) length of a single component region, *N*-hybrid gene, and the proportion of all components contained in *N*-hybrid genes under different length criteria (50 and 100 bp)

Component Length	*N*-hybrid	component region	hybrid gene	ratio
50	2	226(229)	504(473)	0.517(0.539)
	3	494(486)	1196(1275)	0.430(0.407)
	4	680(672)	1275(1275)	0.534(0.527)
	5	766(781)	1275(1275)	0.601(0.613)
	6	922(904)	1275(1275)	0.723(0.709)
100	2	332(316)	514(465)	0.725(0.749)
	3	538(531)	1248(1275)	0.438(0.426)
	4	742(724)	1275(1275)	0.582(0.568)
	5	880(880)	1275(1275)	0.690(0.690)
