# Supplementary material for: Genome-wide detection of hybrid genes with multiple components in human
Source: BMC Res Notes. 2009 May 6;2:75. doi: 10.1186/1756-0500-2-75 (PMC2684099; doi:10.1186/1756-0500-2-75)
Supplement: Additional File 3 — Table S2. The number of events, N-hybrid genes, and component genes of all N-hybrid events with each component length > 50 nucleotides. [file 1756-0500-2-75-S3.pdf]

Table S2. The number of events, *N*-hybrid genes, and component genes of all *N*-hybrid events with each component length > 50 nucleotides

N-hybrid	# of events	# of *N*-hybrid genes	# of component genes
2	438	73	80
3	701	5	36
4	105	1	18
5	34	1	17
6	14	1	11

Note: two *N*-hybrid events are different means either the hybrid gene or at least one of component genes is different from each other.
