# Supplementary material for: Genome-wide detection of hybrid genes with multiple components in human
Source: BMC Res Notes. 2009 May 6;2:75. doi: 10.1186/1756-0500-2-75 (PMC2684099; doi:10.1186/1756-0500-2-75)
Supplement: Additional File 4 — Table S3. List of all mixed Rosetta Stone (MRS) genes and some examples of the involved hybrid events with dual identities. [file 1756-0500-2-75-S4.pdf]

Table S3. List of all mixed Rosetta Stone (MRS) genes and examples of the involved hybrid events with dual identities

MRS genes	Identity	
	Hybrid gene	Component gene
AK131276	AK127911 — AK131276 — AK091126	AK131276<br>AF424542<br>AF194537 AK125782
AK097143	AF194537 — AK097143 — AK095784	AK097143 — NM_001012976 — NM_207471
AK095784	AK127765 — AK095784 — AK124630	AK095784<br>NM_001012976<br>AF258574 AK126238
NM_006267	BX537861 — NM_006267 — CR749330	NM_006267 — CR749330 — NM_005054
NM_032260	BX537861 — NM_032260 — CR749330	NM_032260 — CR749330 — NM_005054
NM_001012976	AK095784<br>NM_001012976<br>AF258574 AK126238	NM_001012976 — AK097143 — AF194537
NM_207471	AK091740 — NM_207471 — AK129962	NM_207471<br>AF424542<br>AK131256 AK095784
AK091740	AK127765<br>AK091740<br>AK124630 AK093664	AK091740 — NM_207471 — AK129962
AK131256	AK127911 — AK131256 — AK091126	AK131256<br>AF424542<br>AK095784 NM_207471
AK131313	AK127911 — AK131313 — AK091126	AK131313 AF194537<br>AF424542<br>AK127847 AK127320
CR749330	NM_006267 — CR749330 — NM_005054	CR749330 — NM_032260 — BX537861
AK125948	AK097920 — AK125948 — AK095757	AK131295<br>AK125948 AF194537<br>AF424542<br>AK127765 AK124630

Note: In simplified notation, we use the *N*-polygon to represent the detected *N*-hybrid events (i.e., *N*-hybrid gene is in the center and connects to the corresponding component genes in *N* vertexes). MRS genes are circumscribed by a rectangle.
